# Evolutionary fine-tuning of residual helix structure in disordered proteins manifests in complex structure and lifetime

**DOI:** 10.1038/s42003-023-04445-6

**Published:** 2023-01-18

**Authors:** Steffie Elkjær, Amanda D. Due, Lise F. Christensen, Frederik F. Theisen, Lasse Staby, Birthe B. Kragelund, Karen Skriver

**Affiliations:** 1 The REPIN and The Linderstrøm-Lang Centre for Protein Science, Department of Biology, University of Copenhagen, Copenhagen, DK-2200 Denmark; 2 Structural Biology and NMR Laboratory, Department of Biology, University of Copenhagen, Copenhagen, DK-2200 Denmark

**Keywords:** Intrinsically disordered proteins, Transcription factors

## Abstract

Transcription depends on complex networks, where folded hub proteins interact with intrinsically disordered transcription factors undergoing coupled folding and binding. For this, local residual structure, a prototypical feature of intrinsic disorder, is key. Here, we dissect the unexplored functional potential of residual structure by comparing structure, kinetics, and thermodynamics within the model system constituted of the DREB2A transcription factor interacting with the αα-hub RCD1-RST. To maintain biological relevance, we developed an orthogonal evolutionary approach for the design of variants with varying amounts of structure. Biophysical analysis revealed a correlation between the amount of residual helical structure and binding affinity, manifested in altered complex lifetime due to changed dissociation rate constants. It also showed a correlation between helical structure in free and bound DREB2A variants. Overall, this study demonstrated how evolution can balance and fine-tune residual structure to regulate complexes in coupled folding and binding, potentially affecting transcription factor competition.

## Abstract (web summary)

Evolution-guided mutagenesis and biophysical analysis reveal that residual helical structure in the binding region of an intrinsically disordered protein regulates the lifetime of its complex by affecting its dissociation.

## Introduction

The structure-function paradigm, linking protein function to 3D structure, is challenged by intrinsically disordered proteins (IDPs) and regions (IDRs), which despite a lack of stable 3D structure can be biologically active^1,2^. IDPs constitute ensembles of conformations^3^, enabling adaptability and complex binding mechanisms^4^. According to the fly-casting mechanism, IDPs benefit from having large capture radii well-suited for target encounter and binding, thus resulting in fast associations^5^. However, binding kinetics for IDPs and globular proteins are not markedly different^6^. Prototypically, IDPs undergo coupled folding and binding^7,8^, and fold before (conformational selection) or after (induced fit) binding. Although some IDPs have been suggested to follow the conformational selection model^9^, most kinetic and structural studies support induced fit^10–14^. Recently, complex interaction patterns, with combined properties from conformational selection and induced fit, are emerging for coupled folding and binding^15–17^. The role of residual helical structure in the free IDP for affinity for target protein interactions is also debated^12,18–20^, and how residual structure in IDPs translates to structure in complexes remains mostly elusive^21^.

Thirty to 40% of eukaryotic proteins contain IDRs longer than 30 residues^22^, and long IDRs are especially prevalent in transcription factors^23^, reflecting key roles of disorder in transcriptional signaling and regulation. Transcription relies on complex signaling mediated by protein–protein interaction networks with highly connected hub proteins as essential nodes. Hub functionality and intrinsic disorder (ID) are highly linked, and several studies have shown folded hubs to interact through short linear motifs (SLiMs) present in IDRs of transcription factors^4,24^. αα-hubs constitute a large group of such folded hubs^25,26^. Among proteins harboring an αα-hub domain are important plant and human transcriptional regulators including Radical-Induced Cell Death1 (RCD1); Sin3; transcription initiation factor TFIID-subunit 4 (TAF4), and CREB binding protein (CBP). αα-hubs are small domains with a common structural composition of 3-5 α-helices decorated on a central αα-hairpin super-secondary structure motif^27^. The organization of the helices relative to the αα-hairpin motif varies between different αα-hub sub-groups, but all αα-hubs expose a hydrophobic ligand-binding cleft^25,26^.

**Fig. 1:**
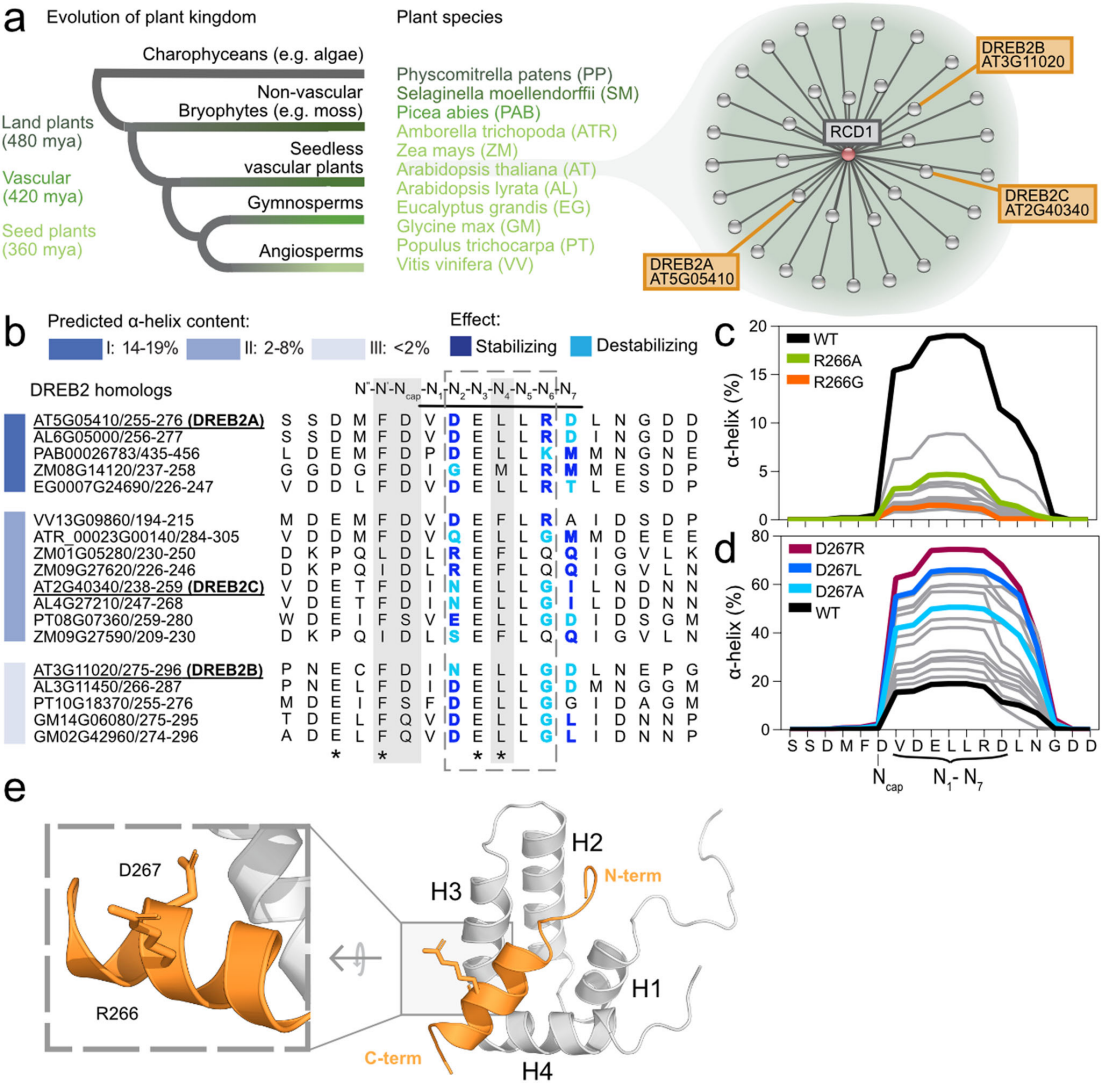
Evolution, interactions, and structure of RCD1 and DREB2s guide selection of DREB2A variants with varying residual helicity. **a** Phylogenetic relationship of the plant kingdom with land plants divided into non-vascular (dark green) and vascular plants, which are further split into seedless (green) and seed plants, finally split into non-flowering gymnosperms (light green) and flowering angiosperms (lightest green). Species are listed in color corresponding to evolution. The *Arabidopsis thaliana* interactome of RCD1 obtained from the STRING database is highlighted to the right with DREB2s indicated. **b** Multiple sequence alignment of the RCD1-RST binding region of DREB2A homologs from different plant species. The aligned region corresponds to *Arabidopsis thaliana* DREB2A(255-272) (AT5G05410) with the RCD1-RST binding region underlined. Based on α-helix (%) predicted by Agadir, the sequences were assigned to three groups: I: 14–19%, II: 2-8%, and III: <2% (blue-colored horizontal bars at left). The α-helical region of free and RCD1-RST-bound DREB2A are marked by the dashed box and the black bar above the alignment, respectively^30^. The position of the hydrophobic stable motif is outlined at top with key stabilizing residues of the N_cap_ boxed in gray and numbering according to N_cap_. Residues in the helical region are colored according to predicted α-helix stabilizing (dark blue) or destabilizing (light blue) effect based on Agadir predictions. SLiM residues are marked with black asterisks. Helicity predictions by Agadir of variants at positions R266 (**c**) and D267 (**d**) in DREB2A with DREB2A-WT in black. All nonaromatic amino acids are included (gray). The substitutions selected for experimental characterization are colored, and DREB2A-D267R is shown in purple. (**e**) HADDOCK model of DREB2A(255-272) (orange) in complex with RCD1-RST(499-572) (silver, PDB: 5oao)^25^ with helix 1-4 (H1-H4) indicated. Left zoom shows surface exposure of D267 and R266 in DREB2A.

RST (RCD1, SRO, TAF4) from RCD1 is a prototypical αα-hub domain and is responsible for most of the known interactions of the plant-specific RCD1^25,28–30^. Knockout of RCD1-RST from the plant model *Arabidopsis thaliana* results in pleiotropic phenotypes such as abnormal shapes of leaves, altered flowering times, and sensitivity to abiotic stresses^28^. The *Arabidopsis* RCD1-RST:transcription factor interaction system constitutes an excellent model for biophysical studies of ID-based interactions. RCD1 has numerous known transcription factor interaction partners with long IDRs^28,30,31^, and the 3D structure of RST and the consensus sequence of the RST-binding SLiM are known^25,30,32^. Furthermore, biophysical studies have demonstrated novel allosteric effects of the SLiM-flanking region on organization of the RST-binding SLiM region^33^. Important RCD1-RST interaction partners include NAM, ATAF1,2, CUC2 (NAC) and dehydration-responsive element-binding protein (DREB)2 transcription factors, present in interactomes that can be traced back to the emergence of land plants (Fig. 1a)^34^. Most αα-hub interactions involve coupled folding and binding^26^, as in the case of DREB2A, which forms a short α-helix when bound to RCD1-RST^25^. Thus, RCD1-RST:DREB2A is a suitable model system for delineating effects of changed residual structure.

With the evolutionarily long tracking of the DREB2A transcription factors, and their documented coupled folding and binding to the RCD1-RST hub domain, we used this system to decipher roles of residual structure in IDPs. By exploiting helix-modulating DREB2A variants, designed based on DREB2 evolution in the RCD1-binding SLiM region, we observed altered binding rate constants with the primary effect of residual structure manifested in complex lifetime. Our analysis also provided insight into the structure in complex, revealing a correlation between helical structure in the free and the bound DREB2A variants. Overall, the study demonstrated how evolution can fine-tune residual helix structure to regulate complex lifetime, a finding of importance to molecular competition.

## Results

### Evolution-guided method for selection of substitutions

To address the role of residual helix structure for ID-based binding and obtain mechanistic insight into the interaction between *Arabidopsis* RCD1-RST and DREB2A, we designed DREB2A(244-272) variants with different amounts of residual helicity in their free state. For this, we took advantage of the interactome of RCD1 and based our choices on evolutionary considerations and structure (Fig. 1). First, structural analysis by nuclear magnetic resonance (NMR) spectroscopy has shown that free DREB2A has a low amount of helical content in the region D262-R266, which increases and expands upon RCD1-RST(499-572)-binding^30^. Second, we predicted the helicity in the RCD1-RST-binding region of DREB2 ligands from phylogenetically representative plant species using Agadir (Fig. 1a–d). The DREB2 ligands could be divided into three groups scored by residual helicity, group I: 14–19%, II: 2–8%, and III: <2% (Fig. 1b). Third, sequence comparison for DREB2 orthologs and paralogs revealed many conserved positions in the SLiM region and in the hydrophobic stable motif^25,35^, including positions N3, N4 and N5 (with N referring to the N_cap_ position), limiting the design of variants.

Based on the evolutionary grouping and the need to maintain conserved positions, we altered the helical content of the region V261-D267 (N1-N7), which forms helix structure in RCD1-RST-bound DREB2A (Fig. 1b)^30^. Searching for positions with varying residues, position N6 of DREB2s of group I differed from those of group II and III by having an overrepresentation of arginine instead of glycine (Fig. 1b). For DREB2C and DREB2B, also from *Arabidopsis* and representing groups II and III, respectively, changing N6 from glycine to arginine increased helicity (Supplementary Fig. 1), suggesting stabilizing and destabilizing effects of arginine and glycine at this position, respectively. N7 is the only position in the helical region where DREB2C and DREB2B differ (Fig. 1b). Predictions for this position showed the isoleucine in DREB2C and the aspartate in DREB2B to have stabilizing and destabilizing effects, respectively (Supplementary Fig. 1). By examining the helix stabilizing effects of the residues present as N6 and N7 (corresponding to R266 and D267 in DREB2A) in the multiple sequence alignment, a pattern with pairs of stabilizing and destabilizing residues appeared (Fig. 1b). N1 was not relevant, as it is consistently occupied by hydrophobic residues without effects on helicity. In contrast, N2 was included in the analysis because of the overrepresentation of stabilizing aspartate residues in group I and III, but not in group II. This evolutionary analysis suggested three positions (D262, R266, and D267 in DREB2A) to regulate the amount of helicity in the RCD1-binding SLiM region of the DREB2 proteins. However, the effect of changing N2 was smaller than that of N6 and N7 (Supplementary Fig. 1), and we therefore focused on N6 and N7.

To increase the helix content of free DREB2A(244-272), D267 was selected, and to decrease the helix content, we aimed at removing the stabilizing effect of R266. Based on predictions (Fig. 1c, d), R266G and D267R were the substitutions with the most extreme effects. As charge reversal is drastic, the substitution predicted to have the second largest effect (D267L) was selected as the stabilizing variant. Importantly, both substitutions, R266G and D267L, are in accordance with the evolutionary-allowed residues at positions N6 and N7. Glycine is found at N6 in almost all DREB2 homologs of group II and III, and leucine is observed at N7 of group III DREB2s (Fig. 1b). According to the RCD1-RST:DREB2A structure model^25^, these positions are surface exposed and not engaged in ligand contacts (Fig. 1e), and therefore likely to only influence folding properties and not binding^36^. In the screen, we noted that alanine substitutions at N6 and N7 had similar, although less, effects on helicity (Fig. 1c, d) and were also selected for experimental characterization, both to represent the more classical approach and to enable trend analysis.

### Ionic-strength dependence of the RCD1-RST:DREB2A interaction

**Fig. 2:**
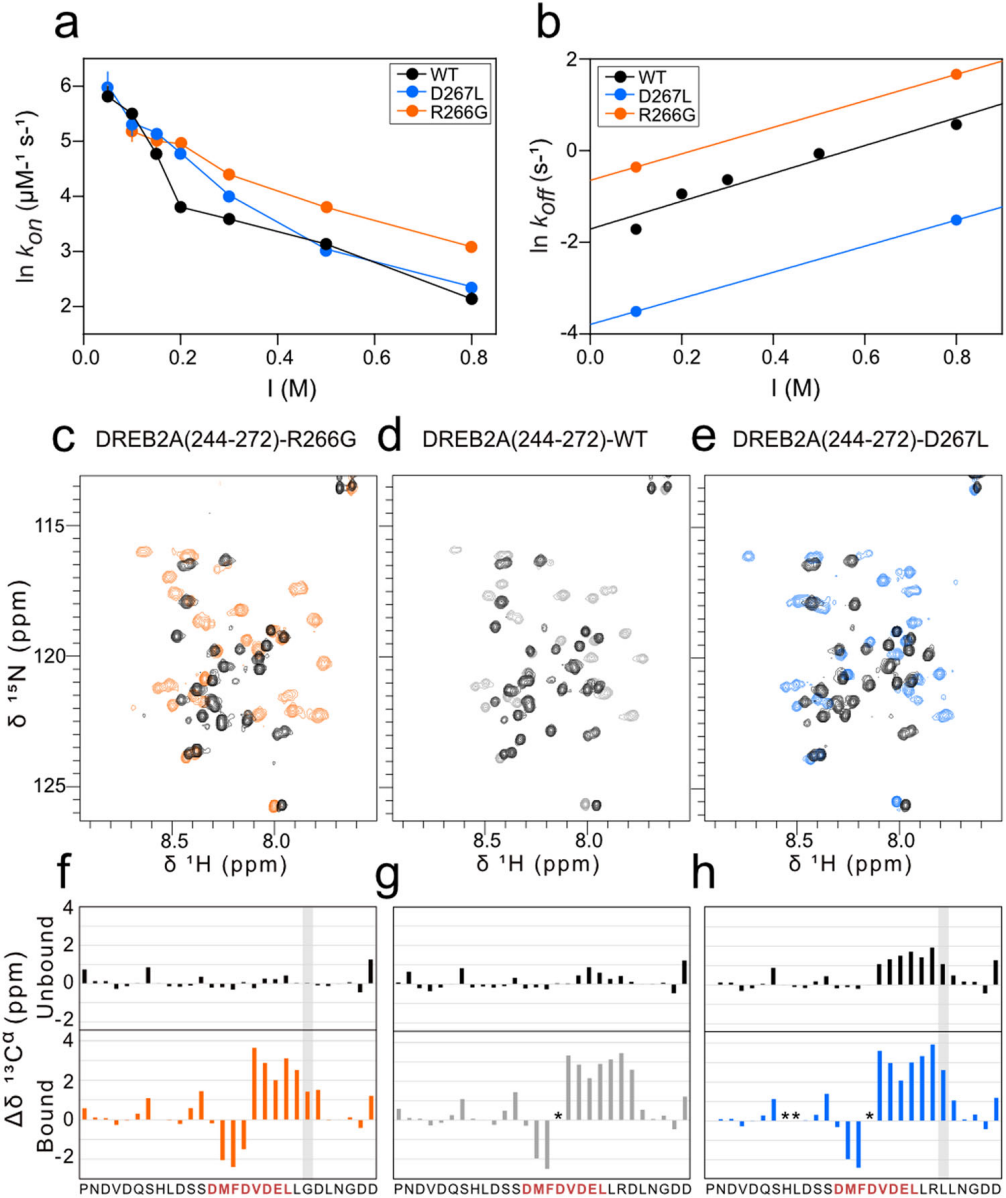
DREB2A(244-272) variants WT, R266G and D267L and their interactions with RCD1-RST(499-572). **a**, **b** Analysis of the role of electrostatics in RCD1-RST(499-572) binding to DREB2A(244-272) variants WT (black), R266G (orange), and D267L (blue) from stopped-flow spectroscopy at 10 °C, 50 mM HEPES pH 7.4, and varying salt concentrations. Error bars represent standard error from fit. Raw data available as Supplementary Data 1. **a** Association kinetics obtained at 50-800 mM NaCl and **b** dissociation kinetics at 100-800 mM NaCl. **c**–**h** Secondary structure analysis of DREB2A variants from NMR analyses with overlayed ^1^H, ^15^N HSQC spectra of ^13^C, ^15^N-labeled DREB2A(244-272)-R266G (**c**), DREB2A(244-272)-WT (**d**), and DREB2A(244-272)-D267L (**e**) in free (black) and RCD1-RST(499-572)-bound states (colored). Secondary C^α^ chemical shifts for **f** DREB2A(244-272)-R266G, **g** DREB2A(244-272)-WT, and **h** DREB2A(244-272)-D267L in its free (upper panel, black) and RCD1-RST(499-572)-bound (lower panel, colored) states. The mutated positions are highlighted (gray bar). The SLiM region (red letters) and residues that could not be assigned (star) are indicated. Raw data available as Supplementary Data 2.

As the modulated residues are charged, we first examined if their substitution, despite surface exposure (Fig. 1e), would lead to undesired effects on binding. Therefore, stopped-flow fluorescence spectroscopy experiments at varying ionic strengths were performed for fluorescently labeled DREB2A(244-272)-R266G, DREB2A(244-272)-D267L and DREB2A(244-272)-WT (Fig. 2a, b; Supplementary Data 1). At 100 mM NaCl, DREB2A(244-272)-WT binding kinetics had already been established with the rate constants *k*_on_ = 240 ± 4 µM^−1^ s^−1^ and *k*_off_ = 0.18 ± 0.01 s^−1^, resulting in a *K*_d_ = 0.8 ± 0.1 nM (Supplementary Fig. 2; Table 1)^37^. When increasing the salt concentration from 50 to 800 mM NaCl, DREB2A(244-272)-WT and DREB2A(244-272)-R266G both displayed a ∼40-fold decrease in *k*_on_ and had similar dependency on ionic strength together with alike basal rate constants, referring to the rate constants in the absence of electrostatic forces (Fig. 2a; Table 2). Thus, the substitution R266G in DREB2A(244-272) did not affect binding through electrostatic effects differently from those of DREB2A(244-272)-WT. We were not able to obtain data for DREB2A(244-272)-D267L at 50 mM NaCl due to noise, and instead compared the ionic strength dependency in the interval of 100–800 mM NaCl. Here, DREB2A(244-272)-D267L displayed a smaller decrease in *k*_on_, meaning less ionic strength dependence, compared to DREB2A(244-272)-WT. However, it still exhibited the same trend in ionic strength dependence with comparable basal association rate constants (Fig. 2a; Table 2). Thus, the substitutions did not affect the ionic strength dependence of *k*_on_. The ionic strength dependency of the dissociation was addressed for DREB2A(244-272)-WT at 100-800 mM NaCl showing a linear relationship and a 9.9-fold increase in *k*_off_ (Fig. 2b; Table 2). The rate constants *k*_off_ for DREB2A(244-272)-R266G and DREB2A(244-272)-D267L exhibited similar changes (7.3 and 7.6-fold, respectively) upon increasing the salt concentration and the changes in overall charge were thus concluded not to influence dissociation (Fig. 2b; Table 2). Altogether, the role of electrostatics did not differ between the DREB2A(244-272) variants, thereby validating our choice of variants. The difference in *k*_on_ at low and high ionic strengths (0.05 vs. 0.8 M) was almost 40-fold (Fig. 2a; Table 2), whereas the difference in *k*_off_ was 10-fold (Fig. 2b; Table 2), indicating that charge-charge interactions are mostly important for association between RCD1-RST(499-572) and DREB2A(244-272).

**Table 1:** Kinetic parameters and *K*_d_ values for DREB2A(244-272) variants binding to RCD1-RST(499-572).

Interaction	*k*_on_ (µM^−1^ s^−1^)	*k*_off_ (s^−1^)	*K*_d_ (nM)
RCD1-RST: DREB2A	240 ± 4^a^	0.18 ± 0.01^a^	0.8 ± 0.1^a^
RCD1-RST: DREB2A-R266G	230 ± 15	0.70 ± 0.01	3.0 ± 0.2
RCD1-RST: DREB2A-R266A	246 ± 15	0.383 ± 0.001	1.5 ± 0.1
RCD1-RST: DREB2A-D267A	222 ± 13	0.07 ± 0.01	0.32 ± 0.06
RCD1-RST: DREB2A-D267L	235 ± 19	0.030 ± 0.001	0.13 ± 0.01

Recorded at 10 °C in 50 mM HEPES, 100 mM NaCl, pH 7.4. Raw data available as Supplementary Data 4.

± Standard error from fit.

^a^From Staby et al.^37^

**Table 2:** Ionic strength dependence of DREB2A(244-272) variants binding to RCD1-RST(499-572).

	*k*_on_ (µM^−1^ s^−1^)			*k*_off_ (s^−1^)		
NaCl	R266G	WT	D267L	R266G	WT	D267L
50 mM	413 ± 102	335 ± 64				
100 mM	206 ± 49	245 ± 11	178 ± 35	0.70 ± 0.01	0.18 ± 0.01	0.030 ± 0.001
150 mM	175 ± 10	118 ± 4	149 ± 19			
200 mM	120 ± 1	45 ± 2	142 ± 9		0.40 ± 0.01	
300 mM	55.9 ± 0.3	36 ± 2	81 ± 3		0.53 ± 0.02	
500 mM	21.0 ± 0.3	23.0 ± 0.2	45 ± 1		0.94 ± 0.01	
800 mM	10.6 ± 0.3	8 ± 1	22 ± 1	5.29 ± 0.05	1.78 ± 0.02	0.223 ± 0.004
Basal rate	6 ± 2	5 ± 2	10 ± 2		5 ± 1	

Recorded at 10 °C in 50 mM HEPES buffer, pH 7.4. Raw data available as Supplementary Data 1.

± Standard error from fit.

### SliM-region substitutions affect helical content in both free and bound forms

**Fig. 3:**
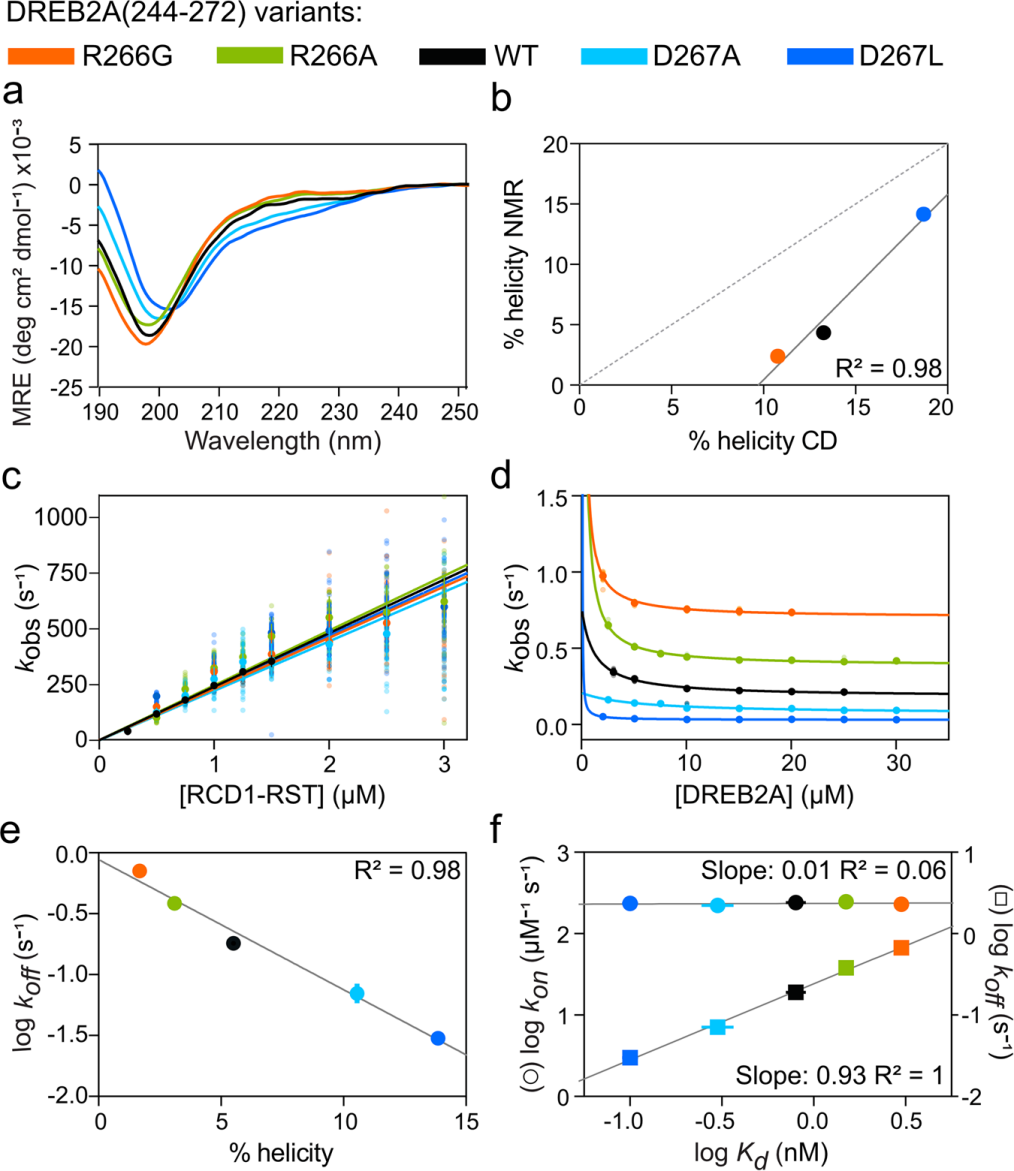
Amount of residual helicity of DREB2A(244-272) variants correlates with complex structure and lifetime. Analysis of DREB2A(244-272)-WT and variants R266G, R266A, D267A, and D267L. **a** Far-UV (190-250 nm) CD spectra of DREB2A variants at 0% (v/v) TFE. All spectra were recorded on 24 µM peptide in 10 mM Na_2_HPO_4_/NaH_2_PO_4_ pH 7.0, 25 °C, and are an average of 10 scans. Raw data available as Supplementary Data 3. **b** Correlation between amount of α-helix in free DREB2A variants measured by NMR and CD spectroscopy. A standard curve was generated by linear regression (Y = 1.538x-14.93). The dashed line represents a perfect 1:1 correlation. **c** Association and **d** dissociation kinetics recorded by stopped-flow fluorescence spectroscopy at 10 °C in 50 mM HEPES pH 7.4, 100 mM NaCl buffer. All individual data points are shown and error bars represent standard deviation from repetitions. Raw data available as Supplementary Data 4. **e** Correlation between dissociation kinetics and α-helix content calculated from the standard curve. **f** LFER plot showing the relationship between *k*_on_ and *k*_off_ and *K*_d_ upon substitution. Linear regression of log *k*_*on*_ and log *k*_off_ is shown. Error bars represent standard error from fit.

Before comparing the effects of the helix modulating substitutions on binding kinetics, we analyzed the content of helix structure by both NMR and circular dichroism (CD) spectroscopy. The overlayed ^1^H,^15^N HSQC spectra of the DREB2A(244-272)-WT and the two variants DREB2A(244-272)-R266G and DREB2A(244-272)-D267L in bound and free states all revealed a higher degree of peak dispersion with upfield shifts in complex with RCD1-RST(499-572), reflecting induction of helical structure upon binding (Fig. 2c–e; Supplementary Data 2). Secondary C^α^ chemical shifts (SCSs) (and ΔδCα – ΔδCβ and δ2Δ for comparison (Supplementary Fig. 3)) were obtained and enabled residue-specific information on the secondary structure content, with consecutive positive and negative values reflecting mostly α-helical and β-sheet/extended structure, respectively^38^. The SCSs confirmed the predicted stabilizing and destabilizing effects of the substitutions, with average SCSs in the region of residual helical structure (D262-R266) of 0.2, 0.5, and 1.6 ppm, corresponding to 6%, 17%, and 51% helix (3.1 ppm used as SCS-reference for 100% helix^39^), for the DREB2A(244-272) variants R266G, WT, and D267L, respectively (Fig. 2f–h; Supplementary Data 2). In complex, all three peptides underwent α-helix stabilization in the region from V261 to D267 with average SCSs at 2.4, 2.9, and 3.1 ppm, resulting in helix content of 79%, 94%, and 100%, for the DREB2A(244-272) variants R266G, WT, and D267L, respectively (Fig. 2f–h). Thus, these data highlight that a correlation (*R*^2^ = 0.70) between helical structure content in the free and the bound DREB2A variants exists. The α-helix content for the N-terminal part of the helix (V261-L264) was similar between DREB2A variants with 91–94% helix. However, the C-terminal part of the helix (L265-D267) contained 58%, 99%, and 107%^39^ helix for the DREB2A(244-272) variants R266G, WT, and D267L, respectively. Trifluoroethanol (TFE) titration experiments, promoting helix formation, were followed by CD spectroscopy and showed that all four DREB2A substitutions affected helix susceptibility, as also predicted by Agadir (Figs. 3a; 1c–d; Supplementary Data 3). The helix content was calculated from the ellipticity at 222 nm as described^40^. In the absence of TFE, 11%, 11%, 13%, 17%, and 19% average helicity was estimated for the DREB2A(244-272) variants R266G, R266A, WT, D267A and D267L, respectively (Supplementary Fig. 4). This order of helix content was maintained when increasing the TFE concentrations from 10 to 70% (Supplementary Fig. 4). Finally, data from NMR and CD were compared by plotting the calculated % helicity based on SCSs vs. the calculated % helicity from CD of the whole peptide (Fig. 3b). These independent data correlated (*R*^2^ = 0.98) and was therefore used to transform CD data of the α-helical content of the alanine variants to comparable content from NMR, resulting in 2, 3, 5, 11, and 14% helix for the DREB2A variants R266G, R266A, WT, D267A, and D267L, respectively. Thus, altogether, the secondary structure analyses supported the order of the predicted helix content in the free state of the variants and furthermore showed the same order of helicity once folded in complex with RCD1-RST.

### DREB2A helix modulating substitutions manifest in altered dissociation rate constants and complex lifetime

To further examine the effects of varying helix content on binding, we performed kinetic experiments on the four DREB2A(244-272) variants for comparison with the data for DREB2A(244-272)-WT (Supplementary Fig. 2, Table 1). The association kinetics of the DREB2A variants were similar to that of the DREB2A(244-272)-WT with *k*_on_ values spanning from 222 ± 13 µM^−1^ s^−1^ to 246 ± 15 µM^−1^  s^−1^ (Fig. 3c; Table 1; Supplementary Data 4). Next, we compared the dissociation kinetics, and the extracted *k*_off_ values were 0.70 ±  0.01 s^−1^, 0.383 ± 0.001 s^−1^, 0.07 ± 0.01 s^−1^, and 0.030 ± 0.001 s^−1^ for the DREB2A(244-272) variants R266G, R266A, D267A, and D267L, respectively (Fig. 3d; Table 1; Supplementary Data 4). The dissociation rate constant of DREB2A(244-272)-WT (*k*_off_ = 0.18 ±  0.01 s^−1^) was in between those of the variants. Furthermore, correlating the dissociation kinetics with the α-helix contents calculated from the secondary structure analysis (Fig. 3e), showed that the higher the content of α-helix in the free state of DREB2A(244-272), the longer the lifetime of the complex with RCD1-RST(499-572) (*R*^2^ = 0.98). The relatively unchanged association kinetics, combined with the altered dissociation kinetics, affected the binding affinity with *K*_d_ values of 3.0 ± 0.2 nM, 1.5 ± 0.1 nM, 0.32 ± 0.06 nM, and 0.13 ± 0.01 nM for the DREB2A(244-272) variants R266G, R266A, D267A, and D267L, respectively (Table 1). Importantly, the affinity of DREB2A(244-272)-WT (*K*_d_ = 0.8 nM) was in between those of the variants. A plot of log *k*_on_ and log *k*_off_ vs. log *K*_d_, known as a linear free energy relationship (LFER), is a valuable method to visualize the contribution of the rate constants to the affinity (Fig. 3f). From the LFER, the slopes reflect the mutual dependency of the affinity and specific rate constants. The LFER of the DREB2A variants emphasized with the horizontal relation between log *k*_on_ and log *K*_d_ (slope 0.007 ± 0.02) that the mutations did not systematically affect association in complex formation. Thus, the altered affinities arose from changed dissociation kinetics (slope of 0.93 ± 0.03) and hence the mutations did not affect the transition state for binding^41^.

### Thermodynamics of DREB2 variants support altered α-helix content and heterogeneity in the complex

We also characterized the thermodynamics of the interactions using isothermal titration calorimetry (ITC). This method allows direct measurement of the affinity (*K*_d_), the change in enthalpy (Δ*H*), and the stoichiometry (N), from where the change in entropy (Δ*S*) and the free energy change (Δ*G*) can be derived. Since the low *K*_d_s for complex formation at 100 mM NaCl and 25 °C did not allow for accurate determination of *K*_d_ by ITC, the measurements were performed in the presence of 300 mM NaCl to decrease affinities (Table 3; Supplementary Data 5). The same order of affinities as determined by kinetics were found, with *K*_d_s of 66 ± 13 nM, 84 ± 9 nM and 369 ± 27 nM for DREB2A(244-272)-D267L, DREB2A(244-272)-WT, and DREB2A(244-272)-R266G, respectively (Supplementary Fig. 5; Table 3). The dramatic decrease in affinity of RCD1-RST(499-572) for DREB2A(244-272)-R266G compared to the helix-stabilizing variant DREB2A(244-272)-D267L and to DREB2A(244-272)-WT was associated with a similar decrease in favorable binding enthalpy, only partly compensated by a less unfavorable change in binding entropy (Table 3). Since the helix content in the free and bound forms of the variants correlated (Fig. 2f–h), the thermodynamics most likely reflected differences in binding optimization (enthalpy) and structural heterogeneity (entropy) in the complex. Hence the helix content in the free state appeared to carry information to both complex structure and thermodynamics.

**Table 3:** Thermodynamics of DREB2A(244-272) variants binding to RCD1-RST(499-572).

Peptide	*K*_d_ nM	*N*	ΔH kJ/mol	-TΔS kJ/mol	ΔG kJ/mol
DREB2A(244-272)-WT	84 ± 9	0.84 ± 0.02	−68 ± 1	27.1	−40.4
DREB2A(244-272)-R266G	369 ± 27	0.85 ± 0.02	−56 ± 1	19.3	−36.9
DREB2A(244-272)-D267L	66 ± 13	0.91 ± 0.09	−69.0 ± 0.9	27.9	−41.1

Recorded by ITC at 25 °C in 50 mM HEPES, pH 7.4, 300 mM NaCl. The standers errors for *ΔH, K*_*d*_ and *N* were obtained from Origin when fitting the data to a model of one set of binding sites. Raw data available as Supplementary Data 5.

The values represent means with *n* = 3 with the corresponding standard error of the mean.

## Discussion

The role of residual structure in IDPs has been debated^12,18–20,42^, but only few studies have analyzed the kinetic effects of modified amounts of residual structure^14,18,20,43^. In such endeavors, alanine variants have traditionally been used^12,19,43^, neglecting the effect of alanine on the ensemble properties. Here, we have developed an orthogonal approach using evolutionary analyses for position selection and substitution design. Inspired by sequence alignments of DREB2 homologs spanning from lower land plants to flowering plants (Fig. 1a)^34^, we found that the amount of residual helix structure within the DREB2 SliM region is balanced to be kept low in the free state, but with propensity for structure induction upon interaction however to a different degree. Two positions in the C-terminal region of the partner-induced helix stand out as responsible for natural balancing of the helical content. To maintain the biological relevance of the system, positions R266 (N6) and D267 (N7) in *Arabidopsis* DREB2A were selected for substitution, including R266G and D267L. DREB2B and DREB2C, which also bind RCD1-RST^28^, contain glycine as N6, and DREB2B has an isoleucine as N7 (Fig. 1b). This indicates that the charges at R266 and D267 in DREB2A are not essential for binding, as also confirmed here, but play other roles. The approach developed here, based on alignment of sequences from evolutionary distant plants, for design of variants assures minimal structural disturbance of the interaction system, a key criterion for molecular comparison.

Studies of IDP variants harboring increased populations of preformed “bound” conformation in the free-state ensemble have shown that increased amount of residual structure correlate with higher binding partner affinity^18–20,42,43^. This was also the case here, and our findings further demonstrated that affinity changes primarily originated from altered dissociation kinetics (Fig. 3; Table 1). Rate constants, *k*_on_s and *k*_off_s obtained for the RCD1-RST:DREB2A interaction were ~10^8 ^M^−1^ s^−1^ and 0.01–1 s^−1^, respectively, and thus in the same range as for other IDP-based systems^41,44^. As for folded proteins, IDPs exhibit a wide range of rate constants, but generally form looser complexes due to higher *k*_off_-values^41^. This agrees with our demonstration that larger content of preformed structure that mimics the bound conformation attenuates dissociation, in accordance with effects on complex lifetime through *k*_off_-values as an emerging trend^12,13^.

Insight into the underlying binding mechanism is important for interpretation of the results obtained in this study. Although the transition state can be affected by mutations in proteins undergoing coupled folding and binding^45,46^, this was not the case for the variants analyzed by LFER in this study (Fig. 3f). The LFER also revealed mechanistic aspects. For conformational selection, increased residual structure would result in a larger *k*_on_ due to a larger fraction of binding competent DREB2A species^11,15,41^. This was not the case for the DREB2A variants (Table 1). The unchanged association kinetics for the most destabilized DREB2A variant, DREB2A(244-272)-R266G (Fig. 3c), suggested that residual structure is not a requirement for association. According to the Smoluchowski equation, the association limit for globular proteins is ∼10^9^–10^10 ^M^−1^ s^−1^ when all collisions result in complex formation^47^. For DREB2A(244-272)-WT, the helical population extracted from SCSs (Fig. 2) suggested that at most 20% of DREB2A forms binding-competent structure. For conformational selection, the number of encounters in the presence of electrostatic interactions should therefore be five times *k*_on_ (5 × 240 = 1200 µM^−1^ s^−1^), which approaches the 10^9^–10^10 ^M^−1^ s^−1^ regime and thereby the limit of possible encounters. At infinite ionic strength the *k*_on_ of DREB2A(244-272)-WT was reduced ∼50-fold compared to its value at 0.1 M salt, and the extracted basal rate constants were also in the range of values defined as non-compatible with the conformational selection pathway (Table 2)^48^. Thus, our results indicate induced fit for the RCD1-RST:DREB2 interaction. However, we cannot exclude that conformational selection may exist, in which case it will account for only a small population.

The affinity of RCD1-RST for the different DREB2A peptides were obtained from both kinetic and thermodynamic analysis (Tables 1 and 3), both demonstrating a correlation between the amount of residual helicity and affinity (*R*^2^ = 0.76 and *R*^2^ = 0.60, respectively) (Supplementary Fig. 6). The relatively low binding affinity determined for DREB2A(244-272)-R266G was due to decreased binding enthalpy, only partly compensated by entropy. This is in accordance with an analysis by Lah et al.^49^, according to which DREB2A(244-272)-R266G would be energetically punished for having less residual structure, but also a higher degree of dynamics in the bound state oppositely reducing the folding penalty. The relation between residual helicity and the amount of formed structure in complex is understudied^21^. We addressed this here, where NMR SCS analyses showed the least helix formation for DREB2A(244-272)-R266G in complex. Thus, despite the induced folding model, structural fine-tuning of the complex was determined by the disordered partner. Together the thermodynamic, kinetic, and structural analyses suggested that a lower content of helical structure in the free state increased disorder in the RCD1-RST:DREB2A complex, easing ligand exchange, and resulting in shortened complex lifetime. Manifestation of residual structure, dynamics, and allosteric regulation in dissociation rate constants were also demonstrated in molecular dynamics simulation studies of the pKID:KIX interaction system^50^ and in several experimental studies of IDPs interacting with folded partners^12–14,51^, pointing to a general model for highly regulated ID-based interaction systems.

**Fig. 4:**
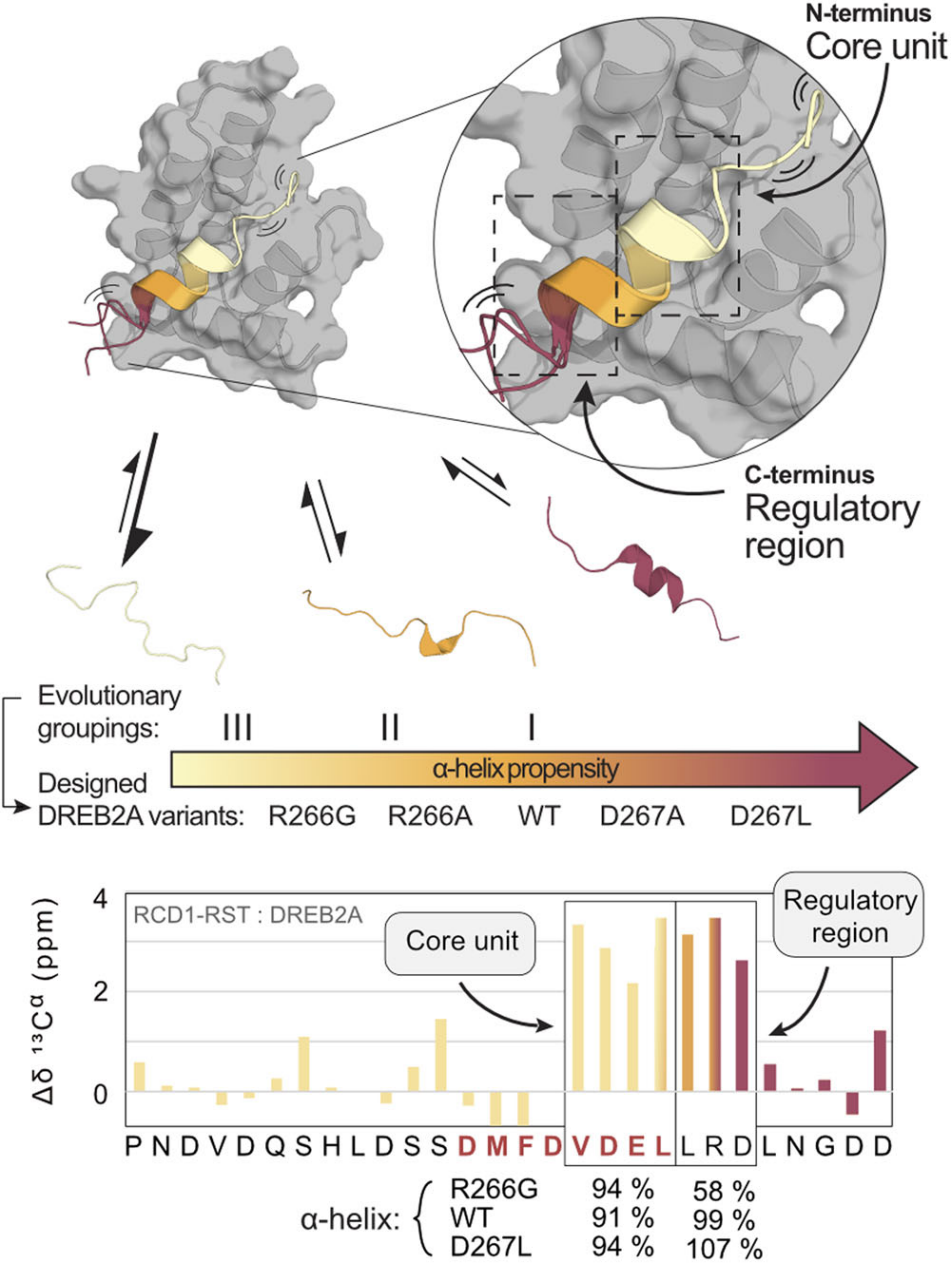
Competitive potential of transcription factor binding to RCD1. Different amounts of residual α-helix structure in the unbound state of transcription factors of the RCD1-interactome result in different affinities and amounts of induced structure upon binding. Here illustrated for both the natural occurring helicity within DREB2 transcription factors and the designed DREB2A variants used in this study. The higher the α-helix amount in the unbound state (color gradient), the higher the affinity, manifested in a smaller *k*_off_ and hence slower off-rates (illustrated by the size of the equilibrium arrows). Higher α-helix amount in the unbound state, is further correlated with a more stabilized C-terminal part of the induced helix. This suggests a core unit and a regulatory region in the bound α-helix. Together, this translates into different competitive potentials of transcription factors binding RCD1.

A recent comparative study of αα-hub domains suggested that the C-terminal region of the *At*RCD1-RST-binding SliM in DREB2A differentially participates in α-helix formation depending on the biological relevance and biochemical specificity of the interaction^52^. With the results obtained here, this jointly promotes a model in which the RCD1-RST-binding region of DREB2A is divided into an N-terminal helix core with a cap defining and stabilizing the induced α-helix and a C-terminal regulatory region with differential folding depending on the partner (Fig. 4). The core folds in all interactions, acting as an anchor. In contrast, the C-terminal region possesses regulatory potential with respect to both selectivity and affinity, and when increasingly dynamic in the bound state it may allow ligand competition access^4^. Based on α-helix propensity, the RCD1-binding region of the DREB2 transcription factors was assigned to three groups (Fig. 1b), reflecting the competitive potential of the stress-associated DREB2s^53^ for interaction with RCD1 (Fig. 4). In the interaction between the tumor suppressor p53 and the E3 ubiquitin ligase Mdm2, designed increased residual helicity in p53 resulted in stronger Mdm2-binding in vitro, and in cells it resulted in impaired target gene expression and failure to induce cell cycle arrest upon DNA damage^19^. In p53, conserved proline residues outside the Mdm2-binding site preserve helix content required for productive p53 signaling^19^. RCD1 negatively regulates its target transcription factors^32,54^, and strong interactions between these and RCD1 could impair plant stress. In the DREB2 transcription factors, the balance between helix stabilizing and destabilizing residues within the RCD1-binding region likely hinders high affinity, long-lived interactions with RCD1, which would have deleterious effects on DREB2 functionality and signaling fidelity. Our work thus suggests that balancing of residual structure can be a powerful tool for fine-tuning complex IDP-based interaction networks.

In conclusion, using an evolution-based design approach we find that residual helical structure in the binding region of an IDP regulates the lifetime of its complex. In detail, the amount of residual helicity in a binding region determines the affinity and translates directly into the degree of structure formation in the bound state. The larger the amount of residual helicity in the free state, the more structured the complex and the longer the lifetime of the complex. Thus, balancing the residual structure may fine-tune interaction networks. This correlation has large implications in understanding disease variants and will, when extrapolated and tested in more systems, provide a tool for design of finely regulated protein–protein interaction networks.

## Methods

### Bioinformatics

For the evolutionary analysis DREB2 homologs (>80 sequences) were obtained from the PLAZA platform^55^. Multiple sequence alignment of these were constructed by Clustal Omega^56^. The *Arabidopsis thaliana* RCD1-interactome was obtained from the STRING database with medium confidence (0.400) and based only on experimental data^57^. Αlpha-helix propensities were predicted by Agadir^58^ with parameters set at pH 7.4, temperature 25 °C, and ionic strength at 0.1. For accessing the structure of RCD1-RST(499-572) and DREB2A(255-272) the HADDOCK model 1 from ref. ^25^ was visualized in PyMOL.

### Peptides and protein expression and purification

RCD1-RST(499-572) was expressed in *E.coli* BL21(DE3) and purified by Source™ 15S (GE Healthcare) cation exchange followed by size exclusion chromatography by Superdex® 75 (Sigma-Aldrich)^25^. The DREB2A(244-272)-WT and variants R266G, R266A, D267A, and D267L were obtained using the QuikChange (Agilent) site-directed mutagenesis kit, after which the GST-tagged peptides were expressed in *E.coli* BL21(DE3) and purified by affinity chromatography with Glutathione Sepharose 4B® resins (GE Healthcare) followed by reversed phase by Vydac® C18^30^. N-terminally fluorescein isothiocyanate (FITC)-labeled and acetylated and C-terminally amidated DREB2A(246-272) peptides were purchased from TAG Copenhagen A/S at >98% purity.

### Nuclear magnetic resonance (NMR) spectroscopy

NMR data were recorded at 25 °C on an AVANCE 800 MHz (^1^H) spectrometer (Bruker) equipped with a cryogenic probe. Samples contained 200 µM ^13^C,^15^N-labeled DREB2A(244-272)-WT or variant as specified, alone or in the presence of 300 µM RCD1-RST(499-572) in 20 mM Na_2_HPO_4_/NaH_2_PO_4_, pH 7.0, 100 mM NaCl with 0.01% (w/v) NaN_3_, 10% (v/v) D_2_O, and 0.7 mM sodium trimethylsilylpropanesulfonate (DSS). Proton chemical shifts were referenced internally to DSS at 0.00 ppm with heteronuclei referenced by relative gyromagnetic ratios. Sets of ^1^H,^15^N HSQC, HNCACB and HNN spectra were recorded for each sample and the backbone chemical shifts were manually assigned. Non-uniformly sampled spectra were reconstructed using qMDD^59^ followed by processing in NMRPipe and analyzed with CcpNMR Analysis^60,61^. The secondary ^13^C^α^ chemical shifts were calculated by subtraction of sequence-dependent random coil values, obtained from the Random Coil Chemical Shifts calculator for IDPs 2 available at SbiNLab (https://www1.bio.ku.dk/english/research/bms/sbinlab/randomchemicalshifts2)^62^, derived from QQXQQ peptide experimental values^63^. A value of 3.1 ppm was used as SCS-reference for 100% helix^39^ and the chemical shifts were further analyzed by δ2Δ^64^ and ΔδCα – ΔδCβ^65^ for comparison.

### Circular dichroism (CD) spectroscopy

Far-UV CD data were recorded on a Jasco-810 spectroscopy with Jasco Peltier temperature control on samples containing 24 µM DREB2A(244-272)-WT or variant in 10 mM Na_2_HPO_4_/NaH_2_PO_4_, pH 7.0, with increasing concentrations of 2,2,2-trifluoroethanol (TFE) (0-70%). Prior to experiments, all samples were spun down at 17,000 × *g* for 10 min. Far-UV CD spectra were recorded between 260 and 190 nm in 1 mm quartz cuvette with parameters set at response time: 4 s, bandwidth: 1.0 nm, data pitch: 0.5 nm, and scanning speed: 50 nm/min. A total of 10 scans were recorded and averaged, from where the identically recorded spectrum of the buffer was subtracted. Mean residue ellipticity (MRE) was calculated from the Eq. 1:

1 $${\left[\theta \right]}_{\lambda }=\frac{{\theta ^\circ }_{\lambda }{{{{{\rm{MRW}}}}}}}{10{lc}}$$

Where the mean residue weight (MRW) was calculated by the molecular weight divided by the number of amino acids, *l* is the path length of the cuvette, and *c* is the concentration given in grams/mL^40^. Helicity was calculated from θ_222_ using Eq. 2:^66^

2 $$\% \,\alpha \,{{{{{\rm{helix}}}}}}=({\left[\theta \right]}_{222}+3000)/(36000+3000)$$

### Isothermal titration calorimetry (ITC)

ITC experiments were performed on a MicroCal™ ITC_200_ microcalorimeter (GE Healthcare). The sample cell contained 9–12 µM DREB2A(244-272)-WT or variant while 92–100 µM RCD1-RST(499-572) was in the syringe. Buffer conditions were 50 mM HEPES, pH 7.4, 300 mM NaCl. Samples were spun down at 17,000 × *g* for 10 min at 25 °C prior to experiments. The first injection was 0.5 µL followed by injections of 2.0 µL with a separation time of 180 seconds, and a total of 18 injection. Water was used as reference and experiments were obtained at 25 °C. ITC data were processed using the Origin 7 software package supplied by the manufacturer and fitted to a one set of sites binding model. The first data point corresponding to the first injection was removed prior to fitting.

### Stopped-flow fluorescence spectroscopy

Stopped-flow fluorescence experiments were performed using a sequential SX20 stopped-flow spectrometer (Applied Photophysics). Fluorescein isothiocyanate (FITC) was used as fluorescence probe and all samples were prepared in 50 mM HEPES buffer, pH 7.4, 100 mM NaCl. The LED lamp of the system had excitation wavelength at 490 nm, and due to the emission peak of FITC at 519 nm, a long-pass cutoff filter at 515 nm was used before recording fluorescence emission above this wavelength. Covalent attachment of FITC to the N-terminus of DREB2A(246-272) did not affect affinity as shown by ITC and the attachment site was outside the RCD1-binding region of DREB2A^33^. Association kinetics were obtained by mixing of equimolar amounts 0.1 µM FITC-DREB2A(246-272) (TAG Copenhagen) and increasing concentrations from 1 to 6 µM RCD1-RST(499-572). Dissociation kinetics were performed as displacement experiments with equimolar mixing of preformed RCD1-RST(499-572):FITC-DREB2A(246-272) complex at concentrations of 1:0.1 µM with increasing concentrations ranging from 5 to 60 µM of un-labeled DREB2A(244-272)-WT and variants. All samples were filtered through a 0.22 µm Q-Max® polyvinylidene difluoride syringe filter (Frisenette, DK) prior to experiments and FITC-samples were handled with aluminum foil to avoid fluorophore bleaching. Stopped-flow fluorescence data were obtained at 10 °C. A total of ∼50 traces were collected and averaged for each independent acquisition. Due to mixing conditions with one reactant in at least 10-fold excess (pseudo-first order conditions), the average trace was fitted to a single exponential equation. The association rate constant (*k*_on_) was determined as the slope of the linear function when plotting the observed rate constants as a function of the varying RCD1-RST(499-572) concentrations. The observed rate constants from the displacement experiments as a function of DREB2A(244-272) concentration were plotted and fitted to a decay function with the asymptote as determination of the dissociation rate constant (*k*_off_).

### Statistics and reproducibility

For stopped-flow data, each independent *k*_obs_ acquisition was made as an average of at least 40 traces fitted to a single exponential equation. Fitting the independent *k*_obs_ values at different concentrations to a linear function (association) or a decay function (dissociation) allows determination of *k*_on_ and *k*_off_ as single experiments (*n* = 1). Thermodynamic parameters were extracted from three independent ITC measurements (*n* = 3). For CD data, each TFE concentration point is a single sample size (*n* = 1) but constituted as the average of 10 scans. No additional statistical analysis was performed.

### Reporting summary

Further information on research design is available in the Nature Portfolio Reporting Summary linked to this article.

## Supplementary information

Supplementary Information

Description of Additional Supplementary Files

Supplementary Data 1

Supplementary Data 2

Supplementary Data 3

Supplementary Data 4

Supplementary Data 5

Reporting summary

## Data Availability

All datasets generated or analyzed during this study are included in this published article (and its supplementary information files). Source data for Figs. 2–3, Tables 1–3, and Supplementary Figs. 2–5 are available as Supplementary Data 1–5.
