# Supplementary material for: Evolutionary fine-tuning of residual helix structure in disordered proteins manifests in complex structure and lifetime
Source: Commun Biol. 2023 Jan 18;6:63. doi: 10.1038/s42003-023-04445-6 (PMC9849366; doi:10.1038/s42003-023-04445-6)
Supplement: Supplementary file 1 — Supplementary Information [file 42003_2023_4445_MOESM1_ESM.pdf]

Supplementary Information

Evolutionary fine-tuning of residual helix structure in disordered proteins manifests in complex structure and lifetime

Steffie Elkjær[1], Amanda D. Due[1,2], Lise F. Christensen[1], Frederik F. Theisen[1,2], Lasse Staby[1,2], Birthe B. Kragelund[1,2,*] and Karen Skriver[1,*]

The [1]REPIN and The Linderstrøm-Lang Centre for Protein Science; [2]Structural Biology and NMR Laboratory, Department of Biology, University of Copenhagen, Copenhagen DK-2200, Denmark

Content:

Supplementary Figure 1: Helicity predictions to guide selection of DREB2A variants
Supplementary Figure 2: Kinetics of the interaction between RCD1-RST(499-572) and DREB2A(244-272)-WT
Supplementary Figure 3: Secondary structure analysis by NMR
Supplementary Figure 4: Amount of residual helicity of DREB2A(244-272) variants is induced upon TFE titration
Supplementary Figure 5: Thermodynamic analysis of RCD1-RST(499-572) and DREB2A(244-272) variants
Supplementary Figure 6: The amount of a-helix in free DREB2A(244-272) variants correlates with the affinity of RCD1-RST(499-572)

Supplementary References

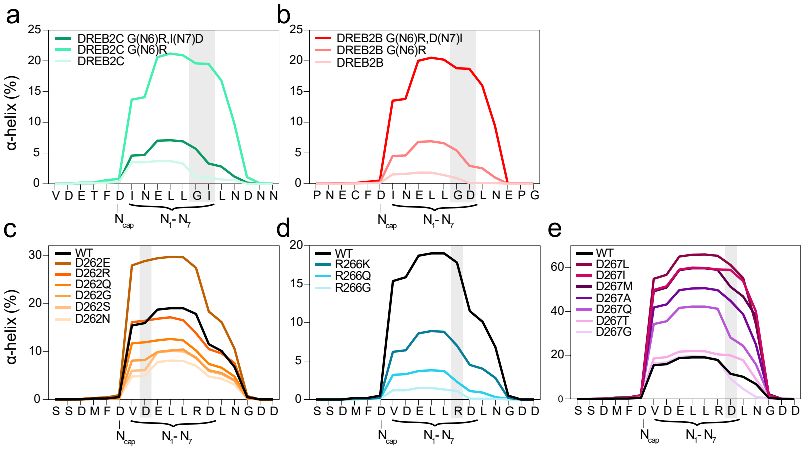

Supplementary Figure 1: Helicity predictions to guide selection of DREB2A variants Helicity predictions by Agadir of the N6 and N7 position in **(a)** DREB2C and **(b)** DREB2B, together with DREB2A and the **(c)** N2, **(d)** N6, and **(e)** N7 position. For **(c-e)** all evolutionary allowed residues were tested.

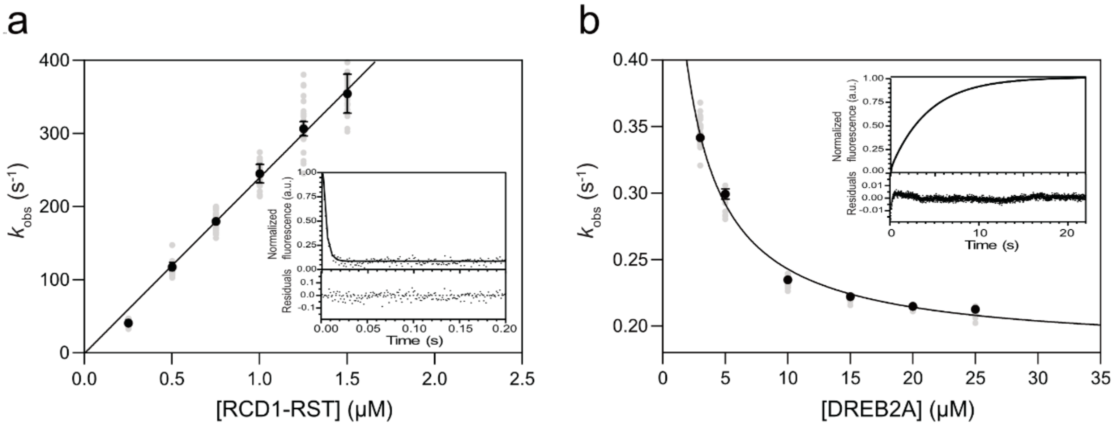

Supplementary Figure 2: Kinetics of the interaction between RCD1-RST(499-572) and DREB2A(244-272)-WT (a) Association and **(b)** dissociation kinetics of DREB2A(244-272)-WT binding to RCD1-RST(499-572) recorded by stopped-flow fluorescence spectroscopy at 10 °C in 50 mM HEPES pH 7.4, 100 mM NaCl buffer. Inserts show typical single exponential fitted kinetic traces with the corresponding residuals below. All individual data points are shown, and error bars represent standard deviation from repetitions. Raw data available as Supplementary Data 4. Data derived from (Staby et al., 2021).

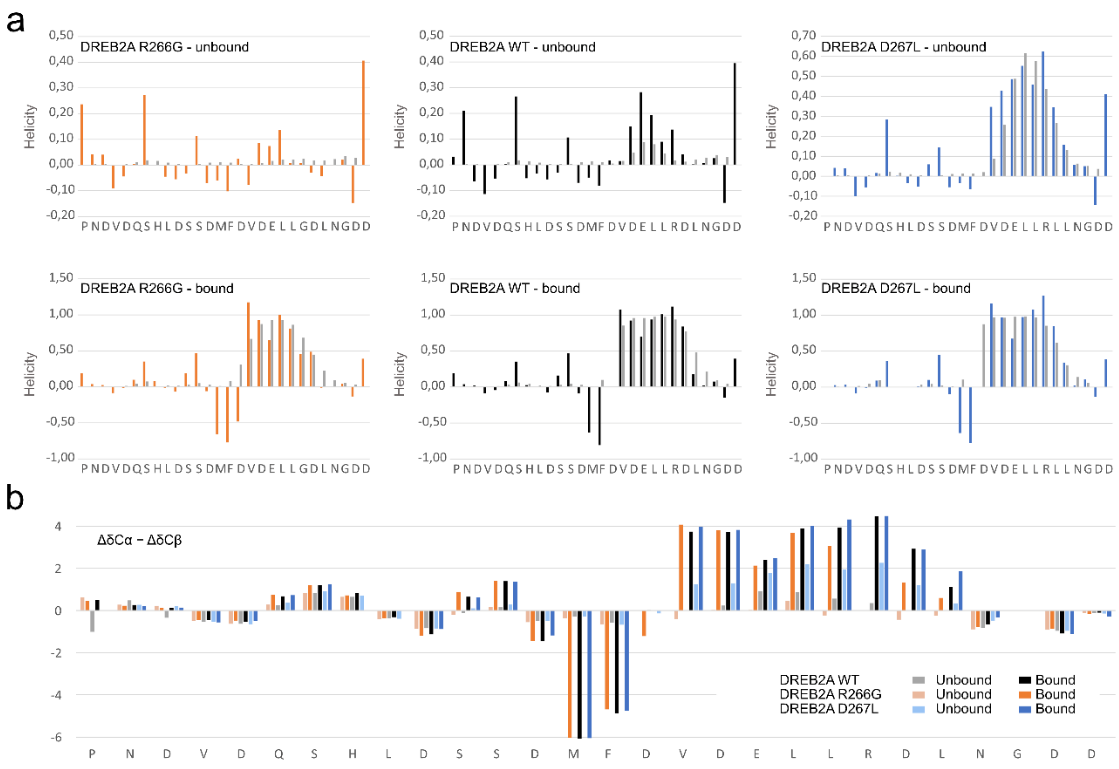

Supplementary Figure 3: Secondary structure analysis by NMR (a) Secondary structure analysis of DREB2A variants with NMR data processed by secondary chemical shifts (SCS) calculations (orange, black, and blue) or by the δ2Δ method (grey). For both, y=1 corresponds to 100% helicity. **(b)** ΔδCα − ΔδCβ values from the SCS analysis. Raw data available as Supplementary Data 2.

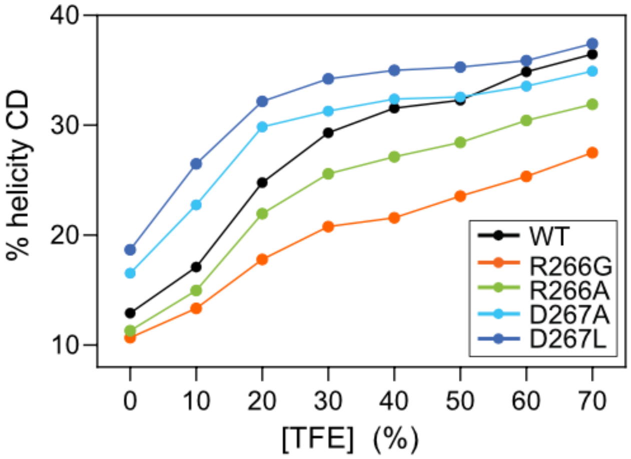

Supplementary Figure 4: Amount of residual helicity of DREB2A(244-272) variants is induced upon TFE titration Analysis of DREB2A(244-272)-WT and variants R266G, R266A, D267A, and D267L. All spectra were recorded on 24 µM peptide in 10 mM Na_2HPO_4/NaH_2PO_4 pH 7.0, 25 °C, and TFE concentrations ranging from 0-70 % (v/v). Each point is an average of 10 scans. Raw data available as Supplementary Data 3.

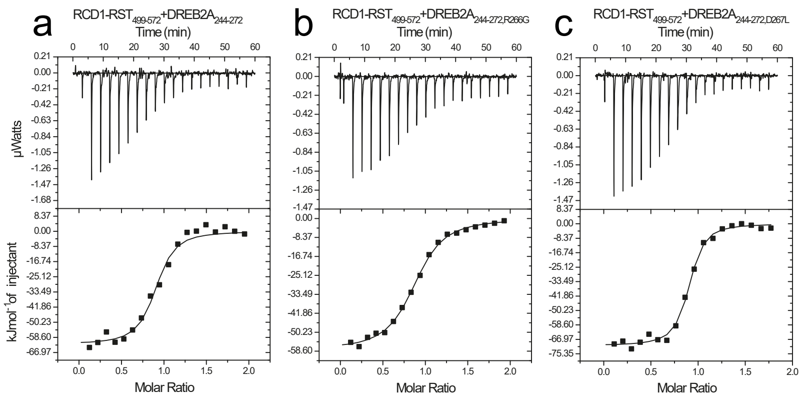

Supplementary Figure 5: Thermodynamic analysis of RCD1-RST(499-572) and DREB2A(244-272) variants Representative ITC isotherms of RCD1-RST(499-572) and DREB2A(244-272) **(a)** WT, **(b)** R266G, and **(c)** D267L. Experiments were recorded at 25 °C in 50 mM HEPES, pH 7.4, 300 mM NaCl and fitted to a one set of binding sites model (solid line). Raw data available as Supplementary Data 5.

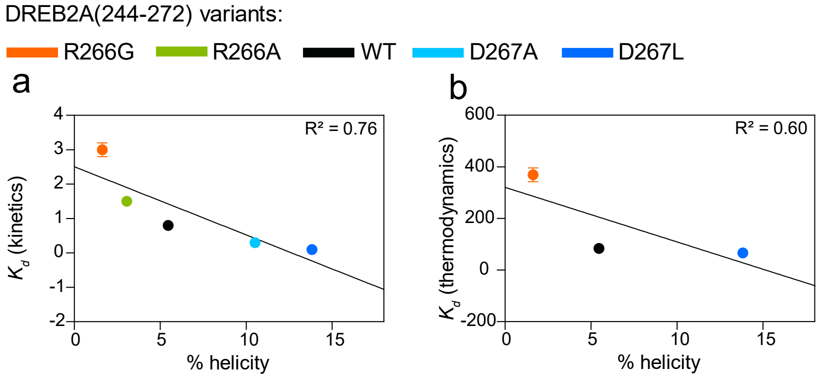

Supplementary Figure 6: The amount of α-helix in free DREB2A(244-272) variants correlates with the affinity of RCD1-RST(499-572) Correlation plot of the amount of residual structure and the affinity obtained by **(a)** kinetics, and **(b)** thermodynamics. Linear regression is shown (line). Kinetics are recorded by stopped-flow fluorescence spectroscopy at 10 °C in 50 mM HEPES pH 7.4, 100 mM NaCl buffer. Thermodynamic experiments were conducted at 25 °C in 50 mM HEPES, pH 7.4, 300 mM NaCl. Error bars represent standard error from fit.

Supplementary References

Staby, L., Due, A. D., Kunze, M. B. A., Jørgensen, M. L. M., Skriver, K., & Kragelund, B. B. (2021). Flanking Disorder of the Folded αα-Hub Domain from Radical Induced Cell Death1 Affects Transcription Factor Binding by Ensemble Redistribution. *Journal of Molecular Biology*, *433*(24), 167320. https://doi.org/10.1016/j.jmb.2021.167320
