# Supplementary material for: Evolutionary fine-tuning of residual helix structure in disordered proteins manifests in complex structure and lifetime
Source: Commun Biol. 2023 Jan 18;6:63. doi: 10.1038/s42003-023-04445-6 (PMC9849366; doi:10.1038/s42003-023-04445-6)
Supplement: Supplementary file 2 — Description of Additional Supplementary Files [file 42003_2023_4445_MOESM2_ESM.pdf]

Description of Additional Supplementary Files

File Name: Supplementary Data 1

Description: Zip.file with Stopped-flow
electrostatic data used for Fig. 2a-b and Table 2

File Name: Supplementary Data 2

Description: Zip.file with NMR data used for
Fig. 2c-h, Fig. 3b and Supp Fig. 3

File Name: Supplementary Data 3

Description: Excel-file with CD data used for
Fig. 3a-b and Supp. Fig. 4

File Name: Supplementary Data 4

Description: Zip.file with Stopped-flow data
used for Fig. 3c-f, Table 1 and Supp. Fig. 2

File Name: Supplementary Data 5

Description: PDF-file with ITC data used for
Table 3 and Supp. Fig. 5
