# Supplementary material for: Evolutionary fine-tuning of residual helix structure in disordered proteins manifests in complex structure and lifetime
Source: Commun Biol. 2023 Jan 18;6:63. doi: 10.1038/s42003-023-04445-6 (PMC9849366; doi:10.1038/s42003-023-04445-6)
Supplement: Supplementary file 7 — Supplementary Data 5 [file 42003_2023_4445_MOESM7_ESM.pdf]

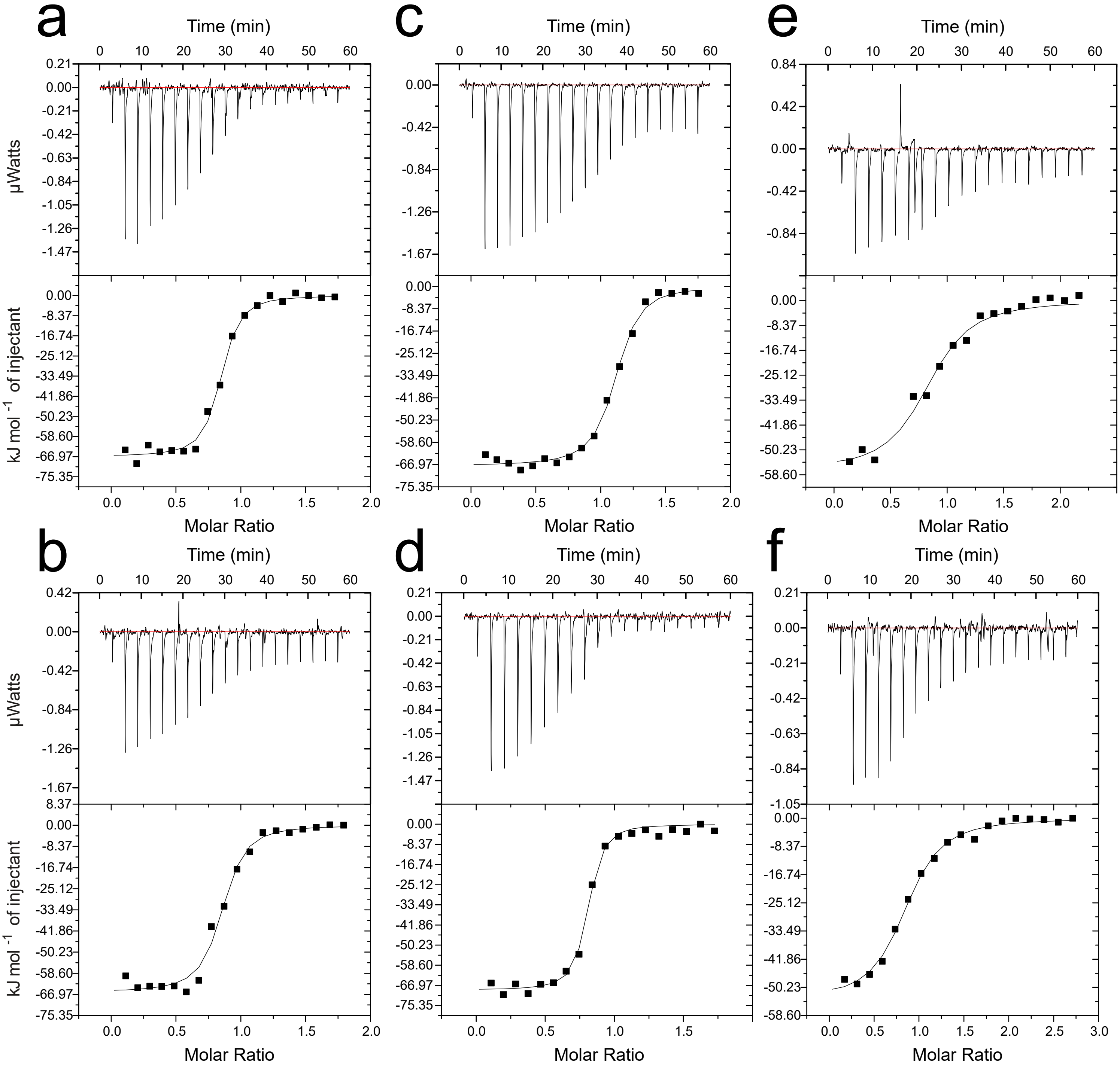


ITC isotherms not already listed in Fig. S5 that constitute the parameters shown in Table 3. Interaction is between RCD1-RST (499-572) and DREB2A (244-272) WT (a-b), D267L (c-d) and R266G (e-f). All experiments is recorded in 50mM Hepes, pH 7.4, 300mM NaCl at 25°C. The data are fitted to a one set of binding site model (solid line).
